# Thermophysical Properties of FUNaK (NaF-KF-UF_4_) Eutectics

**DOI:** 10.3390/ma17112776

**Published:** 2024-06-06

**Authors:** Maxime Fache, Laura Voigt, Jean-Yves Colle, John Hald, Ondřej Beneš

**Affiliations:** 1 European Commission, Joint Research Centre (JRC), 76125 Karlsruhe, Germany; maxime.fache@ext.ec.europa.eu (M.F.); laura.voigt@seaborg.com (L.V.); jean-yves.colle@ec.europa.eu (J.-Y.C.); 2 DTU Construct, Technical University of Denmark, 2800 Kongens Lyngby, Denmark; jhald@dtu.dk; 3 Seaborg Technologies, 2200 Copenhagen, Denmark

**Keywords:** molten salt reactor, NaF-KF-UF_4_, fuel, thermophysical properties, density, vapor pressure, melting point, enthalpy, eutectic, reactor design

## Abstract

General interest in the deployment of molten salt reactors (MSRs) is growing, while the available data on uranium-containing fuel salt candidates remains scarce. Thermophysical data are one of the key parameters for reactor design and understanding reactor operability. Hence, filling in the gap of the missing data is crucial to allow for the advancement of MSRs. This study provides novel data for two eutectic compositions within the NaF-KF-UF_4_ ternary system which serve as potential fuel candidates for MSRs. Experimental measurements include their melting point, density, fusion enthalpy, and vapor pressure. Additionally, their boiling point was extrapolated from the vapor pressure data, which were, at the same time, used to determine the enthalpy of vaporization. The obtained thermodynamic values were compared with available data from the literature but also with results from thermochemical equilibrium calculations using the JRCMSD database, finding a good correlation, which thus contributed to database validation. Preliminary thoughts on fluoride salt reactor operability based on the obtained results are discussed in this study.

## 1. Introduction

Currently, in the nuclear field, a focus exists on the research and development of advanced nuclear reactors [1]. Molten salt reactors (MSRs) constitute a pivotal component of this category, characterized by their utilization of a liquid mixture of inorganic salts as a thermal medium into which nuclear fuel is dissolved [2]. Chloride or fluoride salt mixtures, distinguished by advantageous properties, such as low melting points, high boiling points, and favorable neutronic characteristics, stand out as the most promising candidates for molten salt fuel. Their applicability diverges based on their distinct neutronic absorption properties, with fluorides investigated for thermal reactors and chlorides for fast reactors. For the latter, the isotope ^36^Cl can be formed during reactor operation, from ^35^Cl, by neutron absorption. This isotope can constitute an issue in the case of accidents or leaks due to the high environmental mobility of chlorine. In this regard, fluoride salts are preferable to chloride salts for these applications.

Historically, an emphasis has been placed on beryllium and lithium fluoride salts for thermal molten salt reactors [3]. However, due to the elevated toxicity associated with beryllium, there is an escalating exploration of alternative salts and their properties [4,5,6,7,8]. One such candidate is NaF-KF-UF_4_, commonly denoted as FUNaK, and recent studies have delved into its properties [9,10]. The noteworthy characteristics of FUNaK include the accessibility of all its components, favorable neutronic properties, the absence of the typically required Li enrichment in FLiBe-based salts, and the lower toxicity of Na and K in comparison to Be salts, thereby simplifying research and development as well as waste handling [11]. To mitigate operational temperatures and reduce the strain on materials, the minimization of the melting temperatures of the fuel salt is imperative. Consequently, eutectic compositions are frequently targeted as baseline compositions for molten salt reactors and, more generally, in the field of nuclear reactor coolants [12,13], as they exhibit a local minimum in the liquidus projection of their phase diagram.

The initial phase diagram of NaF-KF-UF_4_ (FUNaK) was presented in a report from 1957 from Oak Ridge National Lab (ORNL) [14]. However, details regarding the procedure for establishing the phase diagram and the potential measurement of the experimental data were not specified. Subsequently, Schacherl et al. thermodynamically assessed the NaF-KF-UF_4_ phase diagram by optimizing the system’s Gibbs energy functions against the phase transition data of 12 novelly measured ternary compositions [9]. This assessment identified the lowest eutectic melting point at 808 K, a result corroborated by experimental DSC data; however, the composition of the eutectic was not experimentally verified. In a recent study by Ocádiz et al. [11] the thermodynamic assessment of the NaF-KF-UF_4_ system was further improved and the compositions of two of FUNaK’s low melting eutectic points were experimentally determined based on a systematic DSC investigation of 25 measurements of various ternary compositions [11]. The two found eutectic compositions are NaF-KF-UF_4_ (55.6-18.7-25.7 mol%) and NaF-KF-UF_4_ (50.4-23.2-26.4 mol%), at melting points of 809 and 810 ± 5 K, similar to what was indicated by Schacherl et al. [9]. Notably, the relatively high content of UF_4_ in these compositions, as opposed to mixtures such as LiF-BeF_2_-ZrF_4_-UF_4_ used in the Molten Salt Reactor Experiment (MSRE) (65.0-29.1-5.0-0.9 mol%), with a 33% U enrichment [3], permits a lower U enrichment to obtain to the same amount of fissile material in the fuel, facilitating the fuel acquisition of more available low-enriched U and regulatory approval.

Data pertaining to the thermophysical and transport properties of FUNaK, including its density, vapor pressure, boiling point, and enthalpies of fusion and vaporization, are notably limited and constitute essential requisites for the development of advanced reactors. The extant information primarily stems from outdated reports originating from the Oak Ridge National Laboratory during the mid-20th century. For instance, Powers et al. collected density results for a range of molten fluoride salt mixtures and additionally compared those to expressions obtained from the molar volume addition formula, which theoretically calculates the densities of mixtures, considering them ideal [15,16,17]. Their findings revealed a deviation of 2% between the calculated and the experimental density for NaF-KF-UF_4_ (46.5-26.0-27.5 mol%) and a maximum deviation of 6% for all of the studied fluoride salt compositions in [16]. In a more recent study, experimental density measurements for NaF-KF-UF_4_ (57.05-16.04-26.91 mol%) were published, indicating a deviation of approximately 2% from the molar volume addition method [10].

The scarcity of existing studies underscores the critical need for updated and comprehensive thermophysical data. Such information is integral to advancing reactor design studies, enhancing the comprehension of fuel behavior during reactor operation, and facilitating risk analyses under both normal and off-normal conditions. This paper presents experimental results encompassing the density, vapor pressure, boiling point, enthalpy of fusion, and enthalpy of vaporization of two eutectic compositions, namely NaF-KF-UF_4_ (55.6-18.7-25.7 mol%) and NaF-KF-UF_4_ (50.4-23.2-26.4 mol%). Additionally, the data are subjected to comparisons with calculations derived from an extensive thermodynamic database (JRCMSD) [18].

## 2. Materials and Methods

The two compositions that we subjected to an experimental determination of their thermophysical properties were:

- NaF-KF-UF_4_ (55.6-18.7-25.7 mol%)—in the further text referred to as FUNaK #1.
- NaF-KF-UF_4_ (50.4-23.2-26.4 mol%)—in the further text referred to as FUNaK #2.

All procedures were executed within argon-filled glove boxes, maintaining O_2_ and H_2_O levels typically below 1 ppm, except for vapor pressure measurements, which were performed in a nitrogen glove box with a significantly reduced oxygen content, i.e., below 4 ppm. We note here that the exposure time of the sample in this glove box was very limited, estimated to be only a few minutes, as the handling only required the direct insertion of the sample prepared in the argon glove box into the device, which was immediately placed under vacuum after insertion.

Both eutectic compositions were prepared by the direct mixing of the constituting end members. Sodium fluoride and potassium fluoride, commercially acquired from Alfa Aesar (Ward Hill, USA), exhibited a certified metal–base purity of 99.99%. Preceding the mixing, NaF and KF underwent heating up to 300 °C in an inert argon environment to evaporate any residual moisture. Uranium tetrafluoride was synthesized in-house utilizing the hydrofluorination method [19]. The purity of the individual components was verified through X-ray diffractometry (XRD), for phase analysis, and differential scanning calorimetry (DSC), for determination of the melting point.

Generally, in the preparation of the salt, individual end members were weighed separately using an analytical scale with an accuracy of ± 0.1 mg. Subsequently, they were ground and mixed in their solid state. Homogenization through pre-melting was carried out as a precursor step before each measurement. The following thermophysical properties were determined for the compositions of FUNaK #1 and FUNaK #2:

- Melting point;
- Density;
- Vapor pressure;
- Boiling point;
- Enthalpy of fusion;
- Enthalpy of vaporization.

The weight of the sample varied for each type of measurement depending on the used method, and the weight range for each property’s determination is reported in Table 1.

### 2.1. Melting Point

The melting points of the FUNaK mixtures were investigated utilizing a SETARAM multi-detector high-temperature calorimeter (Roubaix, France) equipped with a B-type sensor with a peak operational temperature of 1400 °C. As a protective gas, Ar 6.0 (99.9999%+ purity) from Basi (Rastatt, Germany) was used during the measurement at a 15 mL/min flow. To mitigate the potential corrosive vapors emitted by fluorides at elevated temperatures and prevent the interaction of the corrosive molten salt with the stainless steel housing, samples were introduced into a Ni liner, i.e., a cylinder of 22 mm height with a 2.5 mm inner diameter and a wall thickness of 0.1 mm, incorporated within a stainless steel crucible capped with a nickel plate and sealed tightly with a screw-fastened stainless steel lid to ensure a hermetic seal during the measurement [20]. The reference crucible was an empty Ni crucible.

Heating and cooling cycles were repeatedly executed at rates of 10 K/min and 2 K/min to check for result consistency. The measurement consists of an initial cycle serving to homogenize the mixture by melting, which included an isotherm at 1100 °C for 3 h, followed by cooling to room temperature, and two cycles up to 1100 °C at a heating rate of 10 K/min, and one cycle at a heating rate of 2 K/min. The cooling rates were set at 10 K/min. The melting point was based on the onset of the eutectic melting peak of the heat flow curve as, at this point, the solidus is equal to the liquidus. The calibration of the temperature involved the measurement of a series of reference metals from Alfa Aesar (In, Sn, Pb, Zn, Al, Ag) with well-defined melting points, encompassing a substantial portion of the operational temperature range of the calorimeter (from 100 to 1200 °C). A calibration curve, derived from the difference between the measured and actual melting points, was applied to correct the temperatures in all DSC measurements. The temperature difference was attributed to the sensor and sample positions, influenced by the kinetics of heating. Cooling cycles, potentially informing about sample purity or irregular transition phases, were excluded from the melting point determination due to the supercooling effect of halide salts. The comprehensive methodology is explained in the work by Manara et al. [21].

The uncertainty of the determined melting point is ±2 °C, assessed using a systematic uncertainty analysis as the maximum deviation observed between the measured values of the selected reference sample and the literature values.

### 2.2. Fusion Enthalpy

The fusion enthalpy was determined using the same DSC technique as for the melting point’s determination, only in this case a piece of aluminum reference metal in an alumina crucible was inserted in the reference crucible compartment of the DSC as an internal calibrant. Aluminum was chosen as it has a close melting point to the examined FUNaK eutectic samples, but at slightly higher temperatures, so that the signals related to melting do not coincide. From the melting peak of aluminum in the heat flow curve the so-called sensitivity is determined, which gives the relation between the measured signal and the real Joules effect. However, as aluminum melts above the analyzed eutectic points and because the sensitivity of the apparatus changes with temperature, a series of reference metals vs. aluminum calibrations were measured to correct for sensitivity dependence, i.e., to obtain a so-called sensitivity factor, which, at the same time, accounts for the actual geometry of the setup (i.e., two materials measured simultaneously). The selected reference materials used to perform this calibration were Zn, Pb, and Ag, used to obtain a calibration factor valid for a temperature range of 400 °C to 1000 °C, thus wide enough to be relevant for the fusion enthalpy of the two examined eutectic compositions. The reference data of the metals were taken from the literature review by Stolen and Gronvold [22] and the detailed procedure is described in the work of Capelli et al. [23].

An uncertainty of ±2 kJ·mol^−1^ for the enthalpy was determined through a comparison between the experimental and literature enthalpies of the reference materials. Like the melting point analysis, each measurement underwent duplication, and only results from the heating cycles were considered.

### 2.3. Density

Various methods can be used to acquire density measurements in molten salts, such as the bubbling method, the pycnometer, or the loss-in-weight method as described in Magnusson’s review [24]. Herein, the density was measured using the custom-built densitometer detailed in a publication by Beneš et al. [25]. The setup relies on the Archimedes’ principle, using a chemically inert nickel bobber submerged into the liquid salt, housed in a boron nitride crucible. The bobber is affixed to an analytical balance METLER TOLEDO WXTS205DU (with an uncertainty of ±0.1 mg) using a 0.5 mm thick Ni wire spot-welded onto the bobber. The density can be determined with the following equation:

(1) $$\rho =\frac{m_{a}-m_{l}}{V_{i}},$$

in which *ρ* is the density of the molten salt, m_a_ is the apparent mass of the ingot in air, m_l_ is the apparent mass in the molten salt, and V_i_ is the volume of displaced molten salt equivalent to the volume of the Ni ingot. As described in the same paper, the density measurement is corrected for surface tension at the interface between the nickel wire and the liquid salt and for the thermal expansion of the nickel material. For the latter, the nickel density function of temperature was taken from the work of Desai [26]. For the surface tension correction, an additional measurement without the ingot (only the wire submerged into the liquid salt) was conducted and the measured contribution was subtracted from the obtained value using the bobber. The setup was validated by the measurement of the salt mixture LiF-NaF-KF, for which a maximum deviation of 5% was determined compared to the literature.

**Figure 1:**
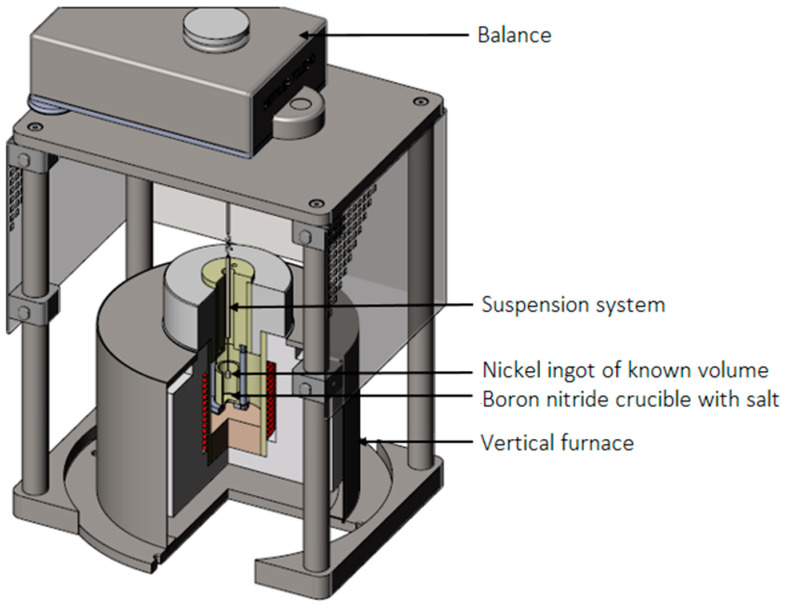
Density setup [25].

The setup for this work is shown in Figure 1, while the material characteristics of the crucible and ingot are outlined in Table 2.

Measurements in argon were taken at room temperature. In the molten salt, measurements were taken in a temperature range from 540 °C to 900 °C. Once the temperature stabilized, and following the stabilization of the scale, five readings of the mass were taken at each temperature. Each composition underwent triplicate measurements to provide an average density trend. Uncertainties were then determined from the quadratic sum of the standard deviations.

### 2.4. Vapor Pressure and Boiling Point

FUNaK’s vaporization behavior was studied using a Knudsen effusion cell coupled with a mass spectrometer (KEMS). This instrument, which has undergone decades of historical development [27], is housed within an alpha-tight and gamma-shielded glove box that meets all the requirements to handle radioactive materials.

Preceding the measurement, the sample and a small tungsten liner containing a silver reference were placed into a Knudsen cell made of tungsten. The thus-prepared Knudsen cell was placed in a furnace made of a tungsten coil covered with a couple of thermal shields. Detailed cell dimensions are outlined in Table 3.

The sample chamber during measurement was maintained under vacuum (~10^−6^ mbar) to avoid interactions between the surrounding atmosphere and evaporated gaseous species from the sample. The emitted gas went through a chopper and a cold trap for condensable residual gas to decrease the background noise. Subsequently, gaseous species were ionized by a cross-beam electron source and detected by a quadrupole mass spectrometer.

For a standard measurement, the sample was heated up to 1500 K, ensuring complete vaporization of the salt at the measurement conditions. The temperature rate was set at 10 K/min and the electron beam was kept constant at 32.8 eV. Experience has shown that the selected ionization energy is optimal to achieve a robust signal for the mass spectrometer. However, it is high enough to induce a dissociation of the gaseous molecules and a correction for such a phenomenon is necessary. This correction is accomplished by measuring its appearance potential at constant temperature, in the current case at 1230 K, selected based on the signal intensity monitored online during the measurement, while increasing the electron energy, typically from 0.5 eV to 40 eV.

The temperature was measured by a pyrometer calibrated using standard materials with well-known melting points, e.g., Zn, Cu, Fe, Ag, Pt, while the electron beam was calibrated using the ionization potential of pure elements, like Ar, Xe, Kr, and He.

Using this technique, the vapor pressure of the different gaseous species p_i_ in equilibrium with condensed matter is obtained through the equation

(2) $$p_{i}=K_{i}\times T\times I_{i}^{+},$$

in which T is the temperature detected by the pyrometer, I_i_^+^ is the signal of the mass spectrometer, and K_i_ is the pressure calibration constant. The last term depends on M_i_, the molar mass of the species; the M_Ag_ of the reference material, i.e., silver for this study; K_Ag_, a calibration factor considering the cell geometry and instrument contributions through reference material signals; and σ_i_ and σ_Ag_, the cross-sections of the species and reference material, respectively. K_i_ is determined by the following equation:

(3) $$K_{i}=\frac{\sqrt{M_{i}}\times \sigma_{\mathrm{Ag}}}{\sqrt{M_{\mathrm{Ag}}}\times \sigma_{i}}\times K_{\mathrm{Ag}}$$

The silver cross-section was determined using the software Sigma (version 1.11, 21/06/90), while the cross-sections of the different end members were calculated using Deutsch’s formula [28]. To calculate this term, the calibration factor K_Ag_ is required and is defined by

(4) $$K_{\mathrm{Ag}}=\frac{p_{\mathrm{Ag}}}{I_{\mathrm{Ag}}^{+}\times T}$$

The Knudsen conditions are fulfilled a few minutes after the start of the measurement, i.e., a Knudsen number Kn > 0.5 with respect to the molecular flow. This asserts that the evaporated molecules flow in one direction without changing their trajectory, which does not affect the equilibrium and consequently makes K_Ag_ constant. p_Ag_ represents the vapor pressure of the silver, computed using the JRCMSD database [18] and based on NIST data [29]; I_Ag_^+^ is the measured intensity of silver from the spectrometer; and T the measured temperature from the pyrometer.

As previously mentioned, a correction for the fragmentation occurring during ionization is imperative to accurately determine the vapor pressure of various gaseous species. It is essential to consider all the corresponding masses for precise vapor pressure data. The appearance potential curve is used to identify the different species and to refine the results.

The vapor pressure determination involves various parameters, each associated with its own uncertainties. The cross-section determination using Deutsch’s equation [28] is commonly cited to have deviations below 20%, and, additionally, some uncertainties related to the temperature in the furnace, here 1%, need to be considered. In a previous study by Beneš et al. [30], an uncertainty of 20% was mentioned when using the same experimental setup. This estimation was derived through a comparison of the ionization energy and vapor pressure of the reference materials, aligning well with our current estimations [30].

## 3. Results

### 3.1. Melting Point

**Figure 2:**
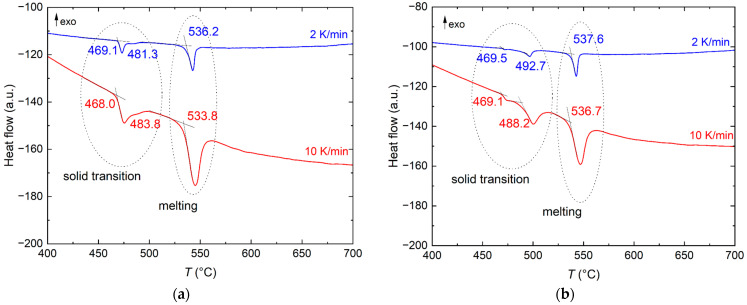
DSC outputs of the heat flow measured for (**a**) FUNaK #1 (NaF-KF-UF_4_ (55.6-18.7-25.7 mol%)) and (**b**) FUNaK #2 (NaF-KF-UF_4_ (50.4-23.2-26.4 mol%)). The heating rates considered are 2 and 10 K/min. The two first peaks correspond to solid-state transitions, while the melting point is determined as the onset temperature of the last peak. Temperature values are corrected. Exothermic transitions are going up, by convention.

The melting points of both FUNaK eutectic compositions were determined by DSC measurements. Figure 2 represents the DSC curves of FUNaK #1 (a) and FUNaK #2 (b), respectively, for a heating rate of 10 K/min (lower red curve) and 2 K/min (upper blue curve). The different 10 K/min heating cycles overlapped largely; hence, only the two last cycles are displayed.

Both compositions exhibit similar melting behavior. A first transition identified at 468.9 ± 2.0 °C, related to Na_3_UF_7_ [9], and a consecutive peak referring to a transition related to NaKUF_6_ [11], i.e., the 1:1:1 phase of FUNaK, are visible [18]. The last transition and, at the same time, the major peak on the DSC heat flow curve correspond to the eutectic melting suggested by the single peak at 2 K/min for both compositions (experience shows that, for certain systems, a slow heating rate better reveals the liquidus shoulder on the heat flow peak). From the average values of the 10 K/min and 2 K/min heating cycles for each composition, the melting point of FUNaK #1 was determined as 535.0 ± 2.0 °C, while, for FUNaK #2, the melting point is 537.2 ± 2.0 °C. This is in line with the experimental findings of Ocádiz et al. [11], who reported melting points at 536 and 537 °C, respectively.

### 3.2. Fusion Enthalpy

Knowledge of the fusion enthalpy of the FUNaK eutectic compositions allows us to quantify the required energy to melt the fuel in the initial phase of reactor operation. From an economic point of view, minimizing this quantity is advantageous as it requires less initial energy input from external resources. Herein, the heat release that occurs when the fuel cools down and solidifies is also important for assessing undercooling scenarios or reactor shutdown in a molten salt reactor.

**Figure 3:**
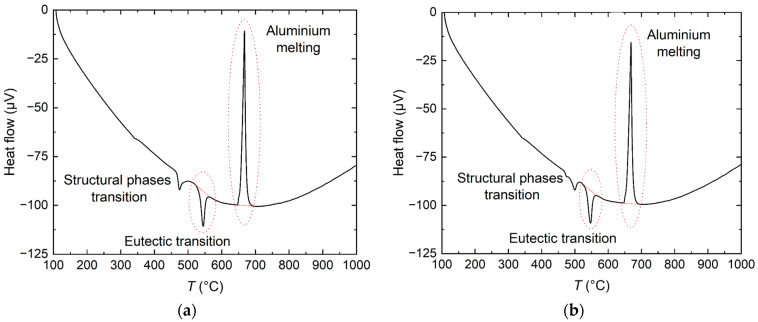
DSC outputs of the measurement of the enthalpy of fusion of (**a**) NaF-KF-UF_4_ (55.6-18.7-25.7 mol%) (FUNaK #1) and (**b**) NaF-KF-UF_4_ (50.4-23.2-26.4 mol%) (FUNaK #2).

Figure 3 represents the DSC graphs of FUNaK #1 and FUNaK #2 for the determination of their fusion enthalpy. Pure aluminum metal was used as a reference and was placed in the empty reference crucible. Using this reference, the heat enthalpy effect was quantified using a temperature rate of 10 K/min. As the reference is the opposite of the sample, the melting peak of the aluminum appears inverted (due to the geometry of the calorimeter), but the nature of the transitions in both crucibles corresponds to endothermic melting transitions.

The obtained results, which were subjected to calibration as discussed in the experimental part of this paper, are reported in Table 4. The resulting fusion enthalpy value is the mean value of four cycles for each measurement. The results for the studied FUNaK #1 and FUNaK #2 compositions are similar, which is expected as their compositions lie close to each other. Furthermore, the table shows a comparison of our obtained values with those reported by Cohen et al. [17] for a somewhat similar composition, showing the same magnitude of the fusion enthalpy.

### 3.3. Density

**Figure 4:**
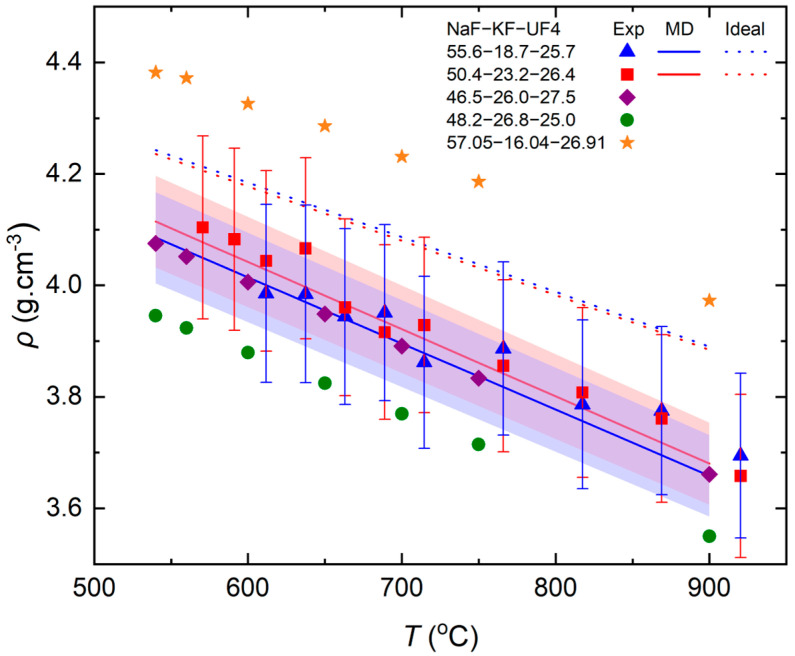
Densities comparison of the NaF-KF-UF_4_ eutectic composition (FUNaK #1) and NaF-KF-UF_4_ eutectic composition (FUNaK #2) in blue triangle dots and red square dots, with the MD model [31] in blue and red lines and the ideal mixture in blue and red dashed lines respectively. Other experimental results from Park et al. [10] in yellow star dots and Cohen and Jones [16] in purple diamond dots and green circle dots for other compositions are plotted for comparison. A 4% uncertainty is considered for the experimental results in this study, while a 2% uncertainty band is displayed for the MD model.

Density data on the liquid fuel are valuable for reactor designs, impacting not only the dimensions but also influencing other thermophysical properties, whilst being crucial for neutronics calculations. Specifically, a denser salt enhances heat conduction through the reactor structure, creating higher thermal conductivity. However, higher density involves extra stresses on the material, in addition to the induced corrosion from the molten salt. Importantly, the thermal expansion of the fuel salt, deduced from density as a function of the temperature data, has an influence on the thermal reactivity coefficient and, hence, the power control of the reactor upon heating and cooling. Understanding the change of density with temperature is a crucial aspect for reactor development. The results are plotted in Figure 4, while Table 5 summarizes the different density equations of the two FUNaK compositions measured in this study. Additionally, the experimental results were compared to the data on slightly different compositions measured by Park et al. [10] and Cohen and Jones [16] and to the density of the ideal mixture from the molecular dynamics (MD) model [31]. Deviant values were obtained close to the melting point, i.e., at 540 °C for FUNaK #1 and at 540, 560, and 580 °C for FUNaK #2, and were omitted for the density correlation.

Uncertainties are determined statistically, by considering the standard deviation related to each measurement conducted in both argon and the molten salt. The measurements demonstrated a maximum deviation of 4% at each temperature, independently of heating or cooling. The last digit of the scale fluctuated and hence an average of several readings was used for each temperature. Further investigations of the measurement’s robustness can refine and lower the determined uncertainty. An uncertainty of 2% was achieved for the MD model [31].

The two measured compositions’ densities, shown in Figure 4, are relatively similar, with an analogous variation with the temperature. The experiments and MD model are in good agreement and lie approximately 4% below the ideal mixture, calculated using the molecular volume addition formula from Powers et al. [15]. Notably, the MD model includes the molecular structure of the melt [11], providing a density estimation that evidentially aligns with the salt’s real behavior (Figure 4).

The obtained novel data on the density of the FUNaK #1 and FUNaK #2 compositions overlap with Cohen and Jones’ data on the close-by NaF-KF-UF_4_ (46.5-26.0-27.5 mol%) composition, also measured based on the Archimedes’ principle [16]. Also, the second composition from Cohen and Jones (48.2-26.8-25.0 mol%) is close to the uncertainty range of the new measurements. As FUNaK #1, FUNaK #2, and the compositions measured by Cohen and Jones [16] are only slightly different compositionally, these small differences are reasonable. This comparison implies that the density was relatively well estimated at the beginning of the 20th century. The composition measured by Park [10] is likewise close to the FUNaK #1 and FUNaK #2 compositions, but its density is significantly higher than this study or Cohen and Jones’ results [16]. The reasons for the deviation of the results between these studies are not clear at this point.

### 3.4. Vapor Pressure

The study of the vapor pressure above the molten fuel is a critical aspect to consider in terms of reactor operability. Volatility may differ among the constituents of the salt, potentially involving changes in the fuel composition over time, preferential releases, or reactions in the gaseous phase, e.g., dimerization, or with the structural material, e.g., plating, pipes’ expansion. As detailed in the experimental section, the measurements using the KEMS setup on selected FUNaK compositions were performed to determine the partial vapor pressures of the mixing components and related molecular species in equilibrium with the molten mixture.

**Figure 5:**
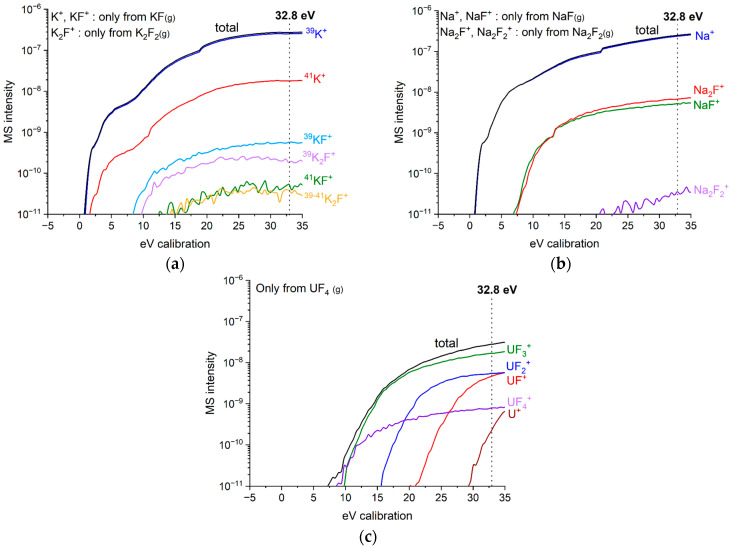
Appearance potentials of end members of FUNaK #2 at T = 1230 K. (**a**) KF; (**b**) NaF; (**c**) UF_4_.

Appearance potential measurements were performed for both FUNaK #1 and FUNaK #2, to correlate the measured signals with the right gaseous species. Figure 5 summarizes the obtained appearance potential results for KF-, NaF-, and UF_4_-related species, respectively. A very similar appearance potential behavior is observed for both eutectic compositions, i.e., the same ionization energy is observed. Tosolin et al. [2] studied the vaporization behavior of UF_4_ and our results correlate well with their work. UF_4,(g)_ undergoes ionization upon electron beam impact, yielding a UF_4_^+^ signal on the spectrometer. Additionally, at the electron energy of the measurement, UF_4(g)_ further dissociates into UF_3_^+^, UF_2_^+^, UF^+^, and U^+^ cations and all these signals contribute collectively to the vapor pressure of UF_4_. Chao [32] studied the vaporization behavior of pure alkali fluorides and detected the presence of dimers of NaF and KF in a non-negligible proportion. Species such as Na^+^, NaF^+^, Na_2_F^+^, and Na_2_F_2_^+^ were identified for the NaF-related species while K^+^, KF^+^, and K_2_F^+^ were observed for the KF-related species. The observed bumps on Figure 5a–c, e.g., at around 20 eV for the ^39^K^+^ signal and Na^+^ signal and around 14 eV for the NaF^+^ and Na_2_F^+^ signals, refer to the change of the instrument’s amplifiers at every new order of magnitude, e.g., from 1 × 10^−10^ values to 1 × 10^−9^ values for the NaF^+^ signal. The appearance potential curve analysis reveals that the K^+^ and KF^+^ signals refer to KF_(g)_ species, while K_2_F^+^ signals refer to K_2_F_2(g)_ species. Following the same analogy as for KF, the Na^+^ and NaF^+^ signals refer to NaF_(g)_ species and the Na_2_F^+^ signals refer to Na_2_F_2(g)_ species.

Additionally, the isotopic ratio of each element needs to be considered. Sodium has only one stable isotope, which was targeted during the measurement. Natural uranium contains 99.2% of its 238 isotope, and its corresponding mass was monitored during the measurement, while the total pressure was corrected to compensate for the remaining isotopes. Potassium is roughly composed of 93% of ^39^K and 7% of ^41^K, and both related species were measured during the experiment. Through the signals obtained experimentally at 32.8 eV, a ratio of 93.3% of ^39^K to 6.7% of ^41^K was determined, similar to the expected isotopic ratio of potassium, confirming the technique.

**Figure 6:**
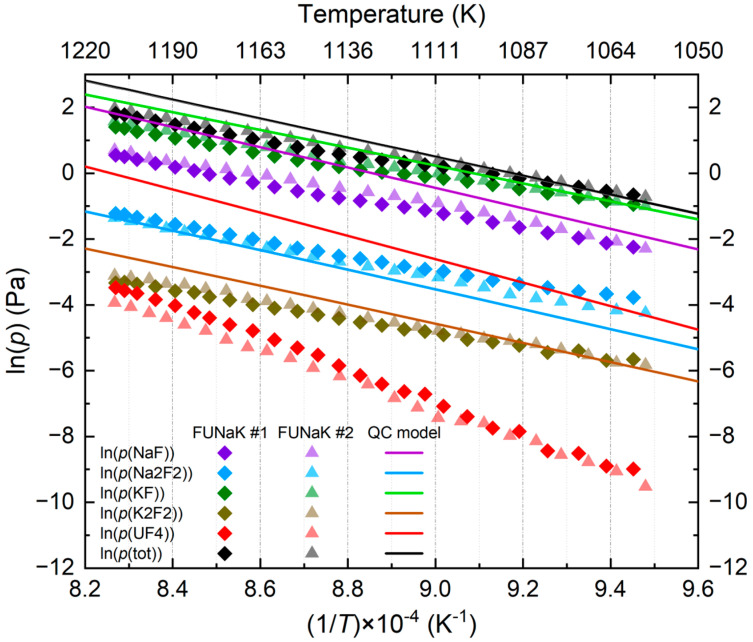
Partial and total vapor pressures of NaF-KF-UF_4_ (55.6-18.7-25.7 mol%)’s composition (FUNaK #1) and of NaF-KF-UF_4_ (50.4-23.2-26.4 mol%)’s composition (FUNaK #2). The total vapor pressure and species vapor pressures for FUNaK #2 calculated from the quasi-chemical (QC) model from Ocádiz et al. [11] are also plotted.

From the appearance potential analysis, the vapor pressures of each species in the salt mixture can be determined, using (2), (3), and (4) listed in the experimental section. Figure 6 represents the measured partial pressures for FUNaK #1 (diamond dots) and for FUNaK #2 (triangle dots) and their associated total vapor pressure. The vapor pressures for all species of both eutectic compositions were calculated from the thermodynamic assessment made by Ocádiz et al. [11] (Figure 6, lines), and it was found that they differ insignificantly between the two compositions. The obtained linearity for all the curves confirms that the Knudsen cell conditions were respected during the measurement, i.e., phase equilibrium was achieved above the homogeneous liquid phase (i.e., no phase transition was involved). Finally, Table 6 groups the different species’ vapor pressure equations. Equation uncertainties were determined through statistical calculations. It is important to note that the liquid FUNaK’s composition at high temperatures slightly differs compared to the low temperature one seen during the measurements due to the preferential vaporization behavior of each species during heating.

The experimental results of the two FUNaK compositions correlate closely. The total vapor pressures are also in good agreement with the total vapor pressure calculated based on the thermodynamic model by Ocádiz et al. [11]. The KF_(g)_ monomer and NaF_(g)_ monomer demonstrate the highest contributions to the vapor phase at the temperatures shown in Figure 6, followed by the Na_2_F_2(g)_ dimer, K_2_F_2(g)_ dimer, and UF_4(g)_. KF_(g)_ is slightly more volatile than NaF_(g)_, whereas the K_2_F_2(g)_ dimer is less volatile than the Na_2_F_2(g)_ dimer. Hence, NaF recombination is more favorable than that of KF, in accordance with the ratio of dimers to monomers of NaF and KF from Chao et al. [32]. UF_4(g)_ has the lowest volatility among all the determined species. Analyzing the vapor pressure equations in Table 6 and the slopes of the vapor pressure data versus temperature in Figure 6, all the species follow a similar vaporization evolution except for UF_4_, whose slope is twice as high as that of the other species. This hints towards an increasing contribution of the partial pressure of UF_4_ to the total vapor pressure at high temperatures.

The comparison of the experimental vapor pressures with those based on the thermodynamic model [11] shows that they are in good agreement with the simulation for most of the species. Only in the case of UF_4_ was a lower partial vapor pressure found compared to the model, which might suggest some degree of inaccuracy in the model in relation to the UF_4_ speciation of the liquid phase.

### 3.5. Boiling Point and the Enthalpy of Vaporization

Knowledge of the boiling point is an additional factor in the safety analysis of a reactor design. This is also true for the knowledge of the enthalpy of vaporization. Both parameters define the safety margins of a reactor and are thus crucial for its operability. The boiling point and enthalpy of vaporization are derived from the vapor pressure data obtained using the KEMS measurements and described in the previous section.

#### 3.5.1. Boiling Point

The boiling point was calculated by extrapolating the partial vapor pressures of various species towards higher temperatures than could be achieved during the measurement. Summing these extrapolated partial vapor pressures, we could identify the temperature point at which the resulting total vapor pressure is equal to atmospheric pressure, which corresponds to the boiling point.

**Figure 7:**
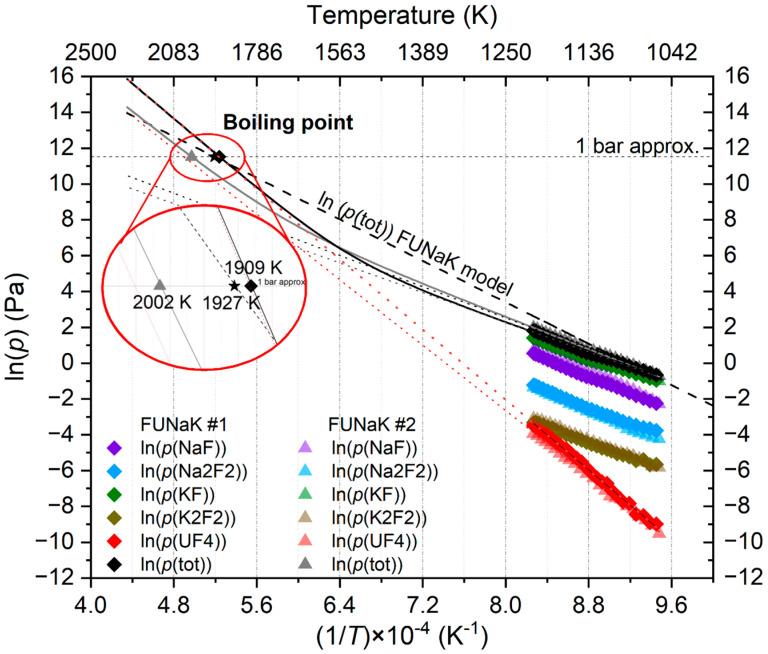
Vapor pressure extrapolation of NaF-KF-UF_4_ (55.6-18.7-25.7 mol%) (FUNaK #1), as a black curved line; NaF-KF-UF_4_ (50.4-23.2-26.4 mol%) (FUNaK #2) from the experiment, as a grey curved line; and of the model based on FUNaK #2’s composition [11], as a dashed black line. The red dashed lines are the linear vapor pressure extrapolation of UF_4(g)_ for FUNaK #1 and FUNaK #2 while the grey dashed lines are the linear total vapor pressure extrapolation for FUNaK #1 and FUNaK #2, showing the higher contribution of UF_4(g)_ at higher temperature. The boiling point, T_B_, determined for the experiments and the FUNaK model are also displayed. T_B_ (FUNaK #1): diamond dot; T_B_ (FUNaK #2): triangle dot; T_B_ (FUNaK model): star dot. In the red circle a zoom of the graph is shown for a better identification of the boiling points.

Figure 7 represents the extrapolated vapor pressure curves obtained for the experimental results for both the examined FUNaK #1 and FUNaK #2 compositions, together with the simulation using the recently assessed thermodynamic model [11] calculated for the FUNaK #2 composition. A separate calculation was carried out for the FUNaK #1 composition, but a nearly identical result was achieved due to the very similar vapor pressures of the two close-lying compositions; therefore, only one line is shown on the graph. Since a relatively large extrapolation was needed to determine the boiling point, a higher uncertainty (±50%) was assigned to the extrapolated vapor pressure compared to the directly measured KEMS data that were subjected to a ±20% error. This corresponds to an uncertainty of ±50 K for the boiling temperature, as discussed further in the discussion section. The results for the boiling points are displayed in Table 7.

The experimental total vapor pressure forms a different curve shape compared to the model, i.e., the experimental extrapolation results in a more convex shape in contrast to the model’s straight line. This curvature is due to the contribution of UF_4_’s vapor pressure, which was found to have a low initial vapor pressure but a higher slope compared to the other end members, such that its contribution to the total vapor pressure is insignificant at low temperatures but dictates it above 1500 K.

#### 3.5.2. Enthalpy of Vaporization

The enthalpy of vaporization, ΔH_vap._, is determined from the measured vapor pressure data using the second law method [2], according to the following formalism:

(5) $$∆H_{\mathrm{vap}.}=-R\frac{d(\ln p_{\mathrm{tot}})}{d(1/T)},$$

in which R is the universal gas constant and $\frac{d(\ln p_{\mathrm{tot}})}{d(1/T)}$ is the slope of the curve ln p_tot_ = f(1/T), which can be defined as follows:

(6) $$\ln p_{\mathrm{tot}}=-\frac{∆H_{T}}{R}\cdot \frac{1}{T}+\frac{∆S_{T}}{R}$$

The enthalpy of vaporization was extrapolated to a temperature range from 298 K to the boiling point, based on the enthalpy of vaporization determined experimentally from the temperature of the experiment T_exp_, which is the average temperature of the KEMS experiment (1230 K). In doing so, the heat capacity of the fuel salt C_p,FUNaK_ (T) was estimated as the weighted average of the end members [9,29], considering their actual molar proportion during the KEMS measurements of both the liquid state and the gas state. The same heat capacities are used to obtain the enthalpy of vaporization of the experiment and the thermodynamic model [11], as displayed in Table 8 and defined as follows:

(7) $$C_{p}\left(T\right)=A+B\times T+C\times T^{2}+D\times T^{3}+E\times T^{4}$$

**Figure 8:**
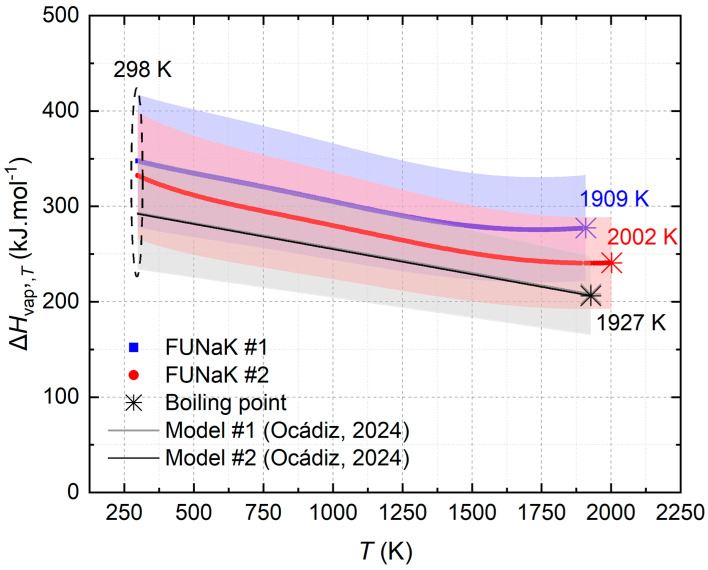
Enthalpy of vaporization evolution with temperature for FUNaK eutectic compositions in blue square dots (FUNaK #1) and in red circle dots (FUNaK #2) and the FUNaK model in gray line and in black line. The dashed black circle indicates the starting temperature for all the curves (T = 298 K). The models for FUNaK #1 [11] and FUNaK #2 [11] are visually the same. An uncertainty of 20% was considered.

The second law analysis was used to extrapolate the enthalpy of vaporization to different temperatures based on the experimental data obtained at T_exp_. Equations (8) and (9) demonstrate how to obtain ΔH_vap,T_ for > T_exp_ > T and T_exp_ < T, respectively. The results are plotted as a function ΔH_vap,T_ = f(T) for the FUNaK eutectic compositions and the model [11], in Figure 8, while the enthalpy of vaporization for T = 298 K, T_exp_, and T_B_, from the experiment and the related curve equations from the model, are mentioned in Table 9. The enthalpy of vaporization was determined from the KEMS measurements and thus the same uncertainty was considered (±20%).

(8) $$\Delta H_{\mathrm{vap},T}\left(\mathrm{FUNaK}\right)+\int_{T}^{T_{\exp}}C_{p,\mathrm{FUNaK},\mathrm{gas}\left(T\right)}\mathrm{dT}=\int_{T}^{T_{\exp}}C_{p,\mathrm{FUNaK},\mathrm{liquid}\left(T\right)}\mathrm{dT}+\Delta H_{\mathrm{vap},T_{\exp}}\left(\mathrm{FUNaK}\right)\mathrm{for}\;T_{\exp}\;>\;T$$

(9) $$\Delta H_{\mathrm{vap},T}\left(\mathrm{FUNaK}\right)+\int_{T_{\exp}}^{T}C_{p,\mathrm{FUNaK},\mathrm{liquid}\left(T\right)}\mathrm{dT}=\int_{T_{\exp}}^{T}C_{p,\mathrm{FUNaK},\mathrm{gas}\left(T\right)}\mathrm{dT}+\Delta H_{\mathrm{vap},T_{\exp}}\left(\mathrm{FUNaK}\right)\mathrm{for}\;T_{\exp}<T$$

The enthalpies of vaporization of FUNaK #1 and FUNaK #2, determined using their experimental vapor pressures, correlate closely, as expected. As for the vapor pressure’s extrapolation, the enthalpy of vaporization curves from the experimental results are differently shaped compared to the model’s straight lines due to the changing gas species contribution of the end members with temperature. Thus, the contribution of each component to the total gaseous heat capacity changes with temperature, leading to curvature. In fact, KF_(g)_ is prevailing in the gas up to 500 K, and above that a combination of KF_(g)_ and NaF_(g)_ results in a slight change of slope of ΔH_vap,T_ at around 500 K, especially for the composition FUNaK #2, which has a higher KF content. From 1500 K onwards, UF_4(g)_ contributes predominantly to the gas mixture. As its gaseous heat capacity is significantly higher than that of the other compounds, the total gas heat capacity approximates linearity, which leads to a progressive stabilization of ΔH_vap,T_ close to the boiling point.

The enthalpies of vaporization of FUNaK #1 and FUNaK #2 are in good agreement with the model, although some discrepancies occur. The model also carries some uncertainties due to error propagation from the extrapolations at lower and higher temperatures. Consequently, the molar ratios of the gas phase of the model are different from the experimental ones, e.g., the molar ratio of NaF is 47 mol% at 1000 K vs. the molar ratio of NaF being 22 mol%, respectively. Thus, the considered enthalpy of vaporization is lower for the model, e.g., around 292 kJ·mol^−1^ at 298 K for both compositions vs. 347.7 and 332.2 kJ·mol^−1^ for experimental FUNaK #1 and FUNaK #2, respectively. The decrease of the value of enthalpy of vaporization with temperature is coherent with a general decrease of energy needed to vaporize the salt at higher temperatures. While FUNaK #1 is closer to the FUNaK model in terms of its boiling point, the model, for the enthalpy of vaporization, is a better approximation of FUNaK #2. Herein, the larger shift of ΔH_vap,T_ between the experiment and model for FUNaK #1 is likely due to the more negative slope of the vapor pressure of UF_4_ in this composition (Figure 6), resulting in a larger deviation from linearity for the total vapor pressure of FUNaK #1, which is not reflected in the model. Nonetheless, the curves lie within the uncertainty range of the experiment and the model, confirming the assumptions made for the heat capacity’s determination, the model developed by Ocádiz et al. [11], and the relevance of the JRCMSD database [18]. Lastly, experimental uncertainties in this section are complicated to improve but provide a good margin for the potential variations of these parameters.

## 4. Discussion

Several thermophysical properties are determined in this paper which are of the utmost importance to the molten salt reactor’s design and to ensure its reliable operation. In this study, two compositions were considered and experimentally ascertained as eutectics. Throughout the study, it was confirmed that both compositions possess similar thermophysical properties, considering the uncertainty margin for each parameter. The melting point determination from this study aligns well with the recent findings of Ocádiz et al. [11], enhancing their data’s reliability. Typically, a eutectic composition is characterized by a distinct and sharp melting transition. It can be argued that the observed onset at ~ 535 °C is not as sharp as expected for a eutectic composition and may hint towards slight deviations of the found compositions from a eutectic composition. However, due to the complexity of the ternary system and an uncertainty in the composition of the measured salt mixtures, a more exact determination of the eutectics is impeded. Notably, even at low heating rates of 2 K/min, only one melting peak, without shoulder, was found, which indicates eutectic melting. Regarding fusion enthalpy, no specific measurement on these eutectic compositions had been performed in the past. Only a comparison with a similar composition, NaF-KF-UF_4_ (46.5-26.0-27.5 mol%), reported by Cohen et al. [17], was feasible and fell within the uncertainty range of (16.2 ± 2) and (17.1 ± 2) kJ·mol^−1^ of our study. The density results demonstrated reasonable agreement with previous data on the named similar compositions from Cohen and Jones [16], falling into the uncertainty range of their measurements of ±5%. Significant differences in density were found compared to the closely related composition of Park, NaF-KF-UF_4_ (57.05-16.04-26.91 mol%) [10], and further comparable studies with the setup of this study and Park’s study, utilizing the same FUNaK composition, may be desirable. Finally, all properties determined by KEMS measurements, i.e., vapor pressure, boiling point, and the vaporization enthalpy of FUNaK, have not yet been investigated in the literature, making the presented results important, novel experimental data for reactor design. The overlap of the results from the KEMS measurements with the estimated values obtained from the modelling work using the thermodynamic model of Ocádiz et al. [11] attests to the reliability of the produced data.

It is reasonable to assume that the investigated eutectic compositions of the FUNaK system in this study are target compositions for engineers building reactors based on FUNaK, due to the melting point minima of these compositions. It is essential to conduct work on thermophysical properties to provide reliable technical data for these fuel salts to aid reactor design and extensively to support safety considerations for nuclear reactor deployment. Based on the data obtained from our study, we conclude the following.

Firstly, the melting point determined during this study is somewhat higher, i.e., ~535 °C, compared to other fuel salt candidates, such as FLiBe-ZrF_4_-UF_4_, which has a melting point of 434 °C [3], implying the need for higher operating temperatures to ensure the salt remains molten throughout the reactor. The risks of thermal cracking due to stress and corrosion are increased with increasing temperatures [33,34,35], and hence higher operating temperatures pose the need for innovative and compatible structural materials. On the other hand, higher operating temperatures lead to more efficient thermal conversion, making the reactor cost effective, which is beneficial for industries; additionally, such a reactor can be considered a direct heat source for non-electricity applications, such as water desalination and heating. The low enthalpy of fusion of FUNaK, i.e., lower than NaCl-PuCl_3_ (64-36 mol%) [36] and some ZrF_4_ salt mixtures [17], requires low power to melt the fuel.

Secondly, a more precise knowledge of the salt’s density, as obtained herein, together with its derivable thermal expansion will facilitate neutronics calculations related to neutron economy, burn-up, and the temperature reactivity coefficient. The density of FUNaK is relatively high compared to U-containing chloride salts or other fluoride salts [3,4,37], necessitating a careful assessment of its impact on the thermal cracking of structural materials. To reduce the risk of this and to investigate the stresses of the salt on the material, multiphysics studies to investigate convection and transport behavior should be performed, necessitating additional measurements of its heat capacity, viscosity, and thermal conductivity, and corrosion studies of FUNaK.

**Figure 9:**
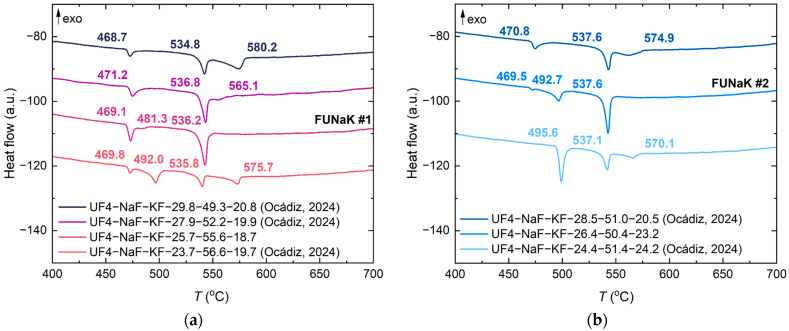
DSC outputs of the heat flow measured for FUNaK compositions (**a**) close to FUNaK #1’s composition (NaF-KF-UF_4_ (55.6-18.7-25.7 mol%)) and (**b**) close to FUNaK #2’s composition (NaF-KF-UF_4_ (50.4-23.2-26.4 mol%)). The considered heating rates are 2 K/min. Exothermic transitions are going up, by convention [11].

Thirdly, the fuel salt’s composition will undergo changes during the lifetime of the reactor through the formation of fission and transmutation products and reactions with structural materials. A shift in the composition will affect the salt’s thermophysical properties, especially its melting point. The change of fuel salt’s composition and the influence of fission product additions on its thermophysical properties was not considered during this study, and this topic will be explored in a future experimental campaign. Figure 9 displays the DSC outputs of FUNaK compositions near two eutectic compositions. For example, when the composition of FUNaK #1 is decreased by 2 mol% in UF_4_ (NaF-KF-UF_4_ (56.6-19.7-23.7 mol%)), an increase in the liquidus to T = 575 °C (offset) is observed. An even higher increase in the liquidus to T = 580 °C is observed when the composition is increased by 4 mol% in UF_4_ (NaF-KF-UF_4_ (49.3-20.8-29.8 mol%)). This temperature shift could occur in real cases through UF_4_ fission. Hence, considering and investigating the effect of deviations of the fuel composition on properties is crucial, and compositional changes during reactor run times should be minimized to avoid the necessity of compensating for the changes, e.g., with higher operating temperatures. Salt vaporization could also be a factor in the change of fuel composition over time. During each density measurement, running for approximately 4 h up to 900 °C, approximately 2% of the salt was lost. Additionally, incongruent evaporation was observed with KEMS, where KF was depleted first, followed by NaF and UF_4_ around the same time. Nevertheless, MSR designs often consider closed or pressurized systems which will inhibit evaporation of the salt. Moreover, refueling can be considered to avoid a change of composition.

The boiling points of FUNaK #1 and FUNaK #2 obtained from the extrapolation of their vapor pressure is relatively high, in the same range as the fuel LiF-ThF_4_-UF_4_ (77.5-20.0-2.5 mol%) considered for the Molten Salt Fast Reactor (MSFR), i.e., 2036 K, estimated by Tosolin et al. [2], and higher than FLiBe, at 1703 K [38]. A difference of 100 K is observed between the boiling point of FUNaK #1 and FUNaK #2. As with their other properties, the boiling point is expected to be similar between the two eutectic compositions due to their comparable compositions and, considering the 50 K uncertainty in the boiling point, some of the difference may be due to error propagation during extrapolation. The evolution of the enthalpy of vaporization plotted in Figure 8 will be useful for reactor safety, to determine the vaporization risk for reactor operability. Due to the large temperature margin between typical operating temperatures of, e.g., ~700 °C and the boiling temperatures of the salt, we suggest that the risk of salt boiling and pressure build-up is low in an overheating scenario. Accident simulations are needed to confirm such assumptions and to set maximum temperature boundaries during incidental scenarios. Importantly, vapor pressure measurements of the fuel salt with fission products will be conducted later to investigate fission product volatility, which is invaluable input for calculating the mechanistic source term and to set up an efficient off-gas system.

Finally, it was demonstrated in the current paper that the thermodynamic model developed by Ocádiz et al. [11], using the JRCMSD database [18], demonstrated good agreements for the boiling point and enthalpy of vaporization, plus its density is well correlated with the molecular dynamics model developed in [31]. From this comparison we can conclude that such simulation tools provide valuable input in assessing thermophysical property data and supporting reactor design.

## 5. Conclusions

Two compositions of the salt NaF-KF-UF_4_ were selected for thermophysical property investigations, identified as experimental eutectic compositions, as outlined in Table 10. For these two compositions, novel thermodynamic data for their melting point, heat of fusion, density, vapor pressures, boiling point, and enthalpy of vaporization were determined. These new thermophysical measurements are crucial for MSR reactor design, filling in the gap of missing data.

The suitability of the two compositions for reactor designs appear roughly equal, given their similar thermophysical properties. Other aspects may be decisive in assessing a designer’s preference for one or the other composition as a reactor fuel, related to, for example, corrosion or fissile material content. Future studies will look into the influence of fission products to the salt on these properties and other properties such as thermal conductivity, viscosity, and heat capacity needed for designing reactors and to strengthen the safety case for this fuel candidate.

## Figures and Tables

**Table 1:** Masses of FUNaK used during the study.

Measurement	Method	FUNaK Mass
Melting point, enthalpy of fusion	DSC	~100 mg
Density	Archimedes’ principle	~6.5 g
Vapor pressure, boiling point, enthalpy of vaporization	Knudsen Effusion MS	~20–30 mg

**Table 2:** Characteristic dimensions of the materials used for the density measurement.

Boron Nitride Crucible		Nickel Ingot	
Dimension	Value (mm)	Dimension	Value (mm)
Inner diameter	12	Ingot diameter	4
Outer diameter	16	Ingot height	6
Height	28	Ni wire diameter	0.5
		Ni wire length	30

**Table 3:** Knudsen cell dimensions used in this work.

Dimension	Value (mm)
Cell height	22
Top hole diameter	0.5
Cell diameter	11

**Table 4:** Experimental fusion enthalpy of several FUNaK compositions.

Sample	Fusion Enthalpy (kJ·mol^−1^)	Reference
NaF-KF-UF_4_ (55.6-18.7-25.7 mol%)	16.7 ± 2.0	This work
NaF-KF-UF_4_ (50.4-23.2-26.4 mol%)	17.1 ± 2.0	This work
NaF-KF-UF_4_ (46.5-26.0-27.5 mol%)	15.7 ± 2.0	[15]

**Table 5:** Density equations as a function of the temperature for FUNaK #1 and FUNaK #2. The average uncertainty of the data is 4%.

	FUNaK #1	FUNaK #2
Ideal mixture	*ρ* (g·cm^−3^) = −0.0010T (°C) + 4.7625	*ρ* (g·cm^−3^) = −0.0010T (°C) + 4.7720
Model	*ρ* (g·cm^−3^) = −0.0012T (°C) + 4.7657	*ρ* (g·cm^−3^) = −0.0012T (°C) + 4.7260
Experiment	*ρ* (g·cm^−3^) = −0.0012T (°C) + 4.8015	*ρ* (g·cm^−3^) = −0.0009T (°C) + 4.5722

**Table 6:** Experimental partial and total vapor pressure equations of NaF-KF-UF_4_ (55.6-18.7-25.7 mol%)’s eutectic composition (FUNaK #1) and NaF-KF-UF_4_ (50.4-23.2-26.4 mol%)’s eutectic composition (FUNaK #2).

Species	Equation	
	FUNaK #1	FUNaK #2
NaF_(g)_	$\ln p(\mathrm{Pa})=-\frac{\left(23292\;\pm \;213\right)}{T\left(K\right)}+\left(19.750\;\pm \;0.188\right)$	$\ln p(\mathrm{Pa})=-\frac{\left(23469\;\pm \;403\right)}{T\left(K\right)}+\left(20.128\;\pm \;0.357\right)$
Na_2_F_2(g)_	$\ln p(\mathrm{Pa})=-\frac{\left(22252\;\pm \;358\right)}{T\left(K\right)}+\left(17.113\;\pm \;0.315\right)$	$\ln p(\mathrm{Pa})=-\frac{\left(24503\;\pm \;221\right)}{T\left(K\right)}+\left(18.872\;\pm \;0.195\right)$
KF_(g)_	$\ln p(\mathrm{Pa})=-\frac{\left(19601\;\pm \;361\right)}{T\left(K\right)}+\left(17.510\;\pm \;0.318\right)$	$\ln p(\mathrm{Pa})=-\frac{\left(20970\;\pm \;145\right)}{T\left(K\right)}+\left(18.873\;\pm \;0.128\right)$
K_2_F_2(g)_	$\ln p(\mathrm{Pa})=-\frac{\left(20961\;\pm \;293\right)}{T\left(K\right)}+\left(14.025\;\pm \;0.258\right)$	$\ln p(\mathrm{Pa})=-\frac{\left(22801\;\pm \;215\right)}{T\left(K\right)}+\;(15.782\;\pm \;0.190)$
UF_4(g)_	$\ln p(\mathrm{Pa})=-\frac{\left(49021\;\pm \;540\right)}{T\left(K\right)}+\left(37.169\;\pm \;0.475\right)$	$\ln p(\mathrm{Pa})=-\frac{\left(45642\;\pm \;419\right)}{T\left(K\right)}+\left(33.864\;\pm \;0.370\right)$
Tot v.p.	$\ln p(\mathrm{Pa})=-\frac{\left(20682\;\pm \;321\right)}{T\left(K\right)}+\left(18.829\;\pm \;0.283\right)$	$\ln p(\mathrm{Pa})=-\frac{\left(21742\;\pm \;117\right)}{T\left(K\right)}+\left(19.922\;\pm \;0.103\right)$
Tot v.p. model [11]	$\ln p(\mathrm{Pa})=-\frac{\left(28993\;\pm \;108\right)}{T\left(K\right)}+\left(26.569\;\pm \;0.073\right)$	$\ln p(\mathrm{Pa})=-\frac{\left(28921\;\pm \;108\right)}{T\left(K\right)}+\left(26.532\;\pm \;0.073\right)$

**Table 7:** Boiling points for the NaF-KF-UF_4_ (55.6-18.7-25.7 mol%) composition (FUNaK #1) and NaF-KF-UF_4_ (50.4-23.2-26.4 mol%) composition (FUNaK #2) from the experiment, and from the model [11], for both compositions.

	Boiling Point T_B_ (K)
FUNaK #1	1909 ± 50
FUNaK #2	2002 ± 50
FUNaK #1 model	1927 ± 50
FUNaK #2 model	1927 ± 50

**Table 8:** Constants for C_p_(T), as defined in (7), used for both FUNaK eutectic compositions and the thermodynamic model [11].

	C_p_	A	B	C	D	E
FUNaK #1	C_p,FUNaK #1,liquid_ (T) (K)	9.895 × 10^1^				
	C_p,FUNaK #1,gas_ (T) (K)	−7.127 × 10^1^	5.298 × 10^1^	−8.515 × 10^−4^	5.409 × 10^−7^	−1.116 × 10^−10^
FUNaK #2	C_p,FUNaK #2,liquid_ (T) (K)	9.961 × 10^1^				
	C_p,FUNaK #2,gas_ (T) (K)	−2.212 × 10^2^	9.390 × 10^−1^	−1.205 × 10^−3^	6.430 × 10^−7^	−1.170 × 10^−10^

**Table 9:** Enthalpy of vaporization for T = 298 K; T_exp_; T_B_**,** for FUNaK eutectic compositions and enthalpy of vaporization equations for the thermodynamic model [11].

	ΔH_vap,T_ (kJ·mol^−1^)		
	298 K	T_exp_	T_B_
FUNaK #1	347.7 ± 69.5	278.6 ± 55.7	277.4 ± 55.5
FUNaK #2	332.3 ± 66.6	247.8 ± 49.6	240.6 ± 48.1
Model #1 [11]	ΔH_vap,T_ (kJ·mol^−1^) = −0.0522 × T (K) + 309.2		
Model #2 [11]	ΔH_vap,T_ (kJ·mol^−1^) = −0.0525 × T (K) + 308.2		

**Table 10:** Summary of the experimental data of this study for the NaF-KF-UF_4_ (55.6-18.7-25.7 mol%) eutectic composition (FUNaK #1) and NaF-KF-UF_4_ (50.4-23.2-26.4 mol%) eutectic composition (FUNaK #2).

Properties	Results	
	FUNaK #1	FUNaK #2
Composition	(55.6-18.7-25.7 mol%)	(50.4-23.2-26.4 mol%)
Molar mass	114.91 g·mol^−1^	117.54 g·mol^−1^
Melting point	(535.0 ± 2.0) °C (808.1 ± 2.0) K	(537.2 ± 2.0) °C (810.3 ± 2.0) K
Heat of fusion	(16.7 ± 2.0) kJ·mol^−1^	(17.1 ± 2.0) kJ·mol^−1^
Liquid density (±5%)	(−0.0012T(°C) + 4.8015) (g·cm^−3^)	(−0.0009T(°C) + 4.5722) (g·cm^−3^)
Boiling point	(1636 ± 50) °C (1909 ± 50) K	(1729 ± 50) °C (2002 ± 50) K
Enthalpy of vaporization T_exp_ (±20%)	(278.6 ± 55.7) kJ·mol^−1^	(247.8 ± 49.6) kJ·mol^−1^

## Data Availability

The data that support the findings of this study are openly available in “Thermophysical properties of NaF-KF-UF4” at https://doi.org/10.5281/zenodo.10911658 [39].

## References

[1] Zohuri B. (2020). Generation IV nuclear reactors. Nuclear Reactor Technology Development and Utilization.

[2] Tosolin A., Beneš O., Colle J.-Y., Souček P., Luzzi L., Konings R.J.M. (2018). Vaporization behaviour of the Molten Salt Fast Reactor fuel: The LiF-ThF_4_-UF_4_ system. J. Nucl. Mater..

[3] Haubenreich P.N., Engel J. (1970). Experience with the Molten-Salt Reactor Experiment. Nucl. Appl. Technol..

[4] Beneš O., Konings R.J.M. (2009). Thermodynamic properties and phase diagrams of fluoride salts for nuclear applications. J. Fluor. Chem..

[5] Sooby E., Baty A., Beneš O., McIntyre P., Pogue N., Salanne M., Sattarov A. (2013). Candidate molten salt investigation for an acclerator driven subcritical core. J. Nucl. Mater..

[6] Williams D.F., Clarno K.T. (2008). Evaluation of Salt Coolants for Reactor Applications. Nucl. Technol..

[7] Beneš O., Konings R.J.M. (2009). Thermodynamic evaluation of the LiF-NaF-BeF_2_-PuF_3_ system. J. Chem. Thermodyn..

[8] Capelli E., Beneš O., Konings R.J.M. (2014). Thermodynamic assessment of the LiF-NaF-BeF_2_-ThF_4_-UF_4_ system. J. Nucl. Mater..

[9] Schacherl B., Eloirdi R., Konings R.J.M., Beneš O. (2021). Thermodynamic Assessment of the NaF-KF-UF_4_ System. Thermo.

[10] Park J., Leong A., Zhang J. (2023). Density Measurements of Molten Salts. J. Chem. Eng. Data.

[11] Ocádiz Flores J.A., Voigt L., Ruszczynski L., Beneš O., Shabar A., Schreuder M., Griveau J.-C., Colineau E., Konings R.J.M., Smith A.L. (2024). Density and viscosity modelling of the NaF-KF-UF_4_ system.

[12] Alekseev P.N., Shimkevich A.L. (2016). Efficacity of eutectic modification of liquid-metal coolants. At. Energy.

[13] Alekseev P.N., Kotov I.A., Shimkevich A.L. (2023). Use of Eutectic Na-Tl Coolant in a Modular Fast Reactor. Phys. At. Nucl..

[14] Thoma R.E., Grimes W.R. (1957). Phase Equilibrium Diagrams for Fused Salt Systems.

[15] Powers W.D., Cohen S.I., Greene N.D. (1963). Physical properties of Molten Reactor Fuels and Coolants. Nucl. Sci. Eng..

[16] Cohen S.I., Jones T.N. (1954). A Summary of Density Measurements on Molten Fluoride Mixtures and a Correlation for Predicting Densities of Fluoride Mixtures.

[17] Cohen S.I., Powers W.D., Greene N.D. (1956). A Physical Property Summary for Fluoride Mixtures.

[18] JRCMSD Database—Thermodynamic Database on Molten Salt Reactor Fuel and Coolant Systems. https://joint-research-centre.ec.europa.eu/joint-research-centre-molten-salt-database-jrcmsd_en.

[19] Souček P., Beneš O., Claux B., Capelli E., Ougier M., Tyrpekl V., Vigier J.-F., Konings R.J.M. (2017). Synthesis of UF_4_ and ThF_4_ by HF gas fluorination and re-determination of the UF_4_ melting point. J. Fluor. Chem..

[20] Beneš O., Konings R.J.M., Wurzer S., Sierig M., Dockendorf A. (2010). A DSC study of the NaNO3–KNO3 system using an innovative encapsulation technique. Thermochem. Acta.

[21] Manara D., Seibert A., Gouder T., Beneš O., Martel L., Colle J.-Y., Griveau J.-C., Walter O., Cambriani A., Blanco O.D. (2020). Experimental methods. Advances in Nuclear Fuel Chemistry.

[22] Stolen S., Gronvold F. (1999). Critical assessment of the enthalpy of fusion of metals used as enthalpy standards at moderate to high temperature. Thermochem. Acta.

[23] Capelli E., Beneš O., Beilmann M., Konings R.J.M. (2013). Thermodynamic investigation of the LiF-ThF_4_ system. J. Chem. Thermodyn..

[24] Magnusson J., Memmott M., Munro T. (2020). Review of thermophysical property methods applied to fueled and un-fueled molten salts. Ann. Nucl. Energy.

[25] Beneš O., Fucina M., Berkmann C., Capelli E. (2024). Density measurements of molten salts.

[26] Desai P. (1987). Thermodynamic properties of Nickel. Int. J. Thermophys..

[27] Colle J.-Y., Freis D., Beneš O., Konings R.J.M. (2013). Knudsen Effusion Mass Spectrometry of Nuclear Materials: Applications and Developments. Electrochem. Soc. Trans..

[28] Deutsch H., Becker K., Basner R., Schmidt M., Mark T.D. (1998). Application of the Modified Additivity Rule to the Calculation of Electron-Impact Ionization Cross Sections of Complex Molecules. Phys. Chem. A.

[29] (2023). NIST Standard Reference Database Number 69.

[30] Beneš O., Capelli E., Vozarova N., Colle J.-Y., Tosolin A., Wiss T., Cremer B., Konings R.J.M. (2021). Cesium and iodine release from fluoride-based molten salt reactor fuel. Phys. Chem. Chem. Phys..

[31] Ruszczynski L., Gheribi A., Silvioli L., Smith A.L. (2024). Molecular dynamics simulation of the structural, thermodynamic and transport properties of the molten salt reactor fuel NaF-KF-UF_4_.

[32] Chao J. (1970). Thermodynamics of vaporization of alkali fluorides. Thermochim. Acta.

[33] Guo S., Zhang J., Wu W., Zhou W. (2018). Corrosion in the molten fluoride and chloride salts and materials development for nuclear applications. Prog. Mater. Sci..

[34] Sridharan K., Allen T. (2013). Corrosion in Molten Salts. Molten Salts Chemistry: From Lab to Applications.

[35] Allen T., Busby J., Meyer M., Petti D. (2010). Materials challenges for nuclear systems. Mater. Today.

[36] Karlsson T.Y., Middlemas S.C., Nguyen M.-T., Woods M.E., Tolman K.R., Glezakou V.-A., Hermann S.D., Schorne-Pinto J., Johnson D., Reddish S.E. (2023). Synthesis and thermophysical property determination of NaCl-PuCl_3_ salts. J. Mol. Liq..

[37] Moon J., Andrews H., Agca C., Bilheux J.-C., Braatz A., McAllister A., McFarlane J., McMurray J., Robb K., Zhang Y. (2022). Density measurements of various molten Sodium, Magnesium, Potassium, and Uranium Chloride salt compositions using Neutron Imaging. IEC Res..

[38] Zheng G., He L., Carpenter D., Sridharan K. (2016). Corrosion-induced microstructural developments in 316 stainless steel during exposure to molten Li2BeF4(FLiBe) salt. J. Nucl. Mater..

[39] Fache M., Colle J.-Y., Beneš O. (2024). Thermophysical Properties of NaF-KF-UF_4_.

